# An Essential Role for Zygotic Expression in the Pre-Cellular Drosophila Embryo

**DOI:** 10.1371/journal.pgen.1003428

**Published:** 2013-04-04

**Authors:** Zehra Ali-Murthy, Susan E. Lott, Michael B. Eisen, Thomas B. Kornberg

**Affiliations:** 1 Cardiovascular Research Institute, University of California San Francisco, San Francisco, California, United States of America; 2 Department of Molecular and Cell Biology, University of California Berkeley, Berkeley, California, United States of America; 3 Howard Hughes Medical Institute, University of California Berkeley, Berkeley, California, United States of America; The University of North Carolina at Chapel Hill, United States of America

## Abstract

The Drosophila embryo proceeds through thirteen mitotic divisions as a syncytium. Its nuclei distribute in the embryo's interior during the first six divisions, dividing synchronously with a cycle time of less than ten minutes. After seven divisions (nuclear cycle 8), the syncytial blastoderm forms as the nuclei approach the embryo surface and slow their cycle time; subsequent divisions proceed in waves that initiate at the poles. Because genetic studies have not identified zygotic mutants that affect the early divisions and because transcription has not been detected before cycle 8, the early, pre-blastoderm embryo has been considered to rely entirely on maternal contributions and to be transcriptionally silent. Our studies identified several abnormal phenotypes in live *engrailed* (*en*) mutant embryos prior to cycle 8, as well as a small group of genes that are transcribed in embryos prior to cycle 7. Nuclei in *en* embryos divide asynchronously, an abnormality that was detected as early as nuclear cycle 2–3. Anti-En antibody detected nuclear En protein in embryos at cycle 2, and expression of an En:GFP fusion protein encoded in the paternal genome was also detected in cycle 2 nuclei. These findings demonstrate that the Drosophila embryo is functionally competent for gene expression prior to the onset of its rapid nuclear divisions and that the embryo requires functions that are expressed in the zygote in order to faithfully prosecute its early, pre-cellularization mitotic cycles.

## Author Summary

Genetic studies identified many genes that are required during Drosophila oogenesis to endow the embryo with structures and components it will need to develop; they have also identified many genes that the embryo must express. However, measures of transcription have detected zygotic transcripts only after seven nuclear divisions, and many studies have concluded that zygotic mutants do not affect embryos prior to cellularization. The model that has emerged is that the earliest stages of embryogenesis rely solely on maternal stores and do not receive input from the zygotic genome. The fact that the embryo's nuclei divide rapidly with a cycling time of less than ten minutes has been interpreted to support this model, because it has been assumed that the nuclear cycle is too short for productive gene expression. Using sensitive measures of transcription and histological procedures that detect subtle differences, we found evidence for expression as early as nuclear cycle 2, and we identified a requirement for zygotic gene expression in embryos with just 2–4 nuclei. These findings challenge the idea that the Drosophila embryo is entirely pre-programmed and that its early development is under exclusive maternal control.

## Introduction

Drosophila embryogenesis is remarkably rapid, precise and reproducible. In its first two-three hours, thirteen syncytial nuclear divisions distribute approximately 6,000 nuclei around the periphery of the embryo. These divisions are rigidly choreographed, and although little is known of the mechanisms that regulate them, it has been generally accepted that they are entirely programmed during oogenesis and are independent of information encoded in the genome of the zygote. This notion is based on several factors. First, the early nuclear cycles are less than ten minutes, making productive gene expression seem improbable. Second, transcription has not been detected prior to nuclear cycle eight [1]–[8]. And third, whereas genetic studies have identified many maternal-effect functions that are required during oogenesis to support the nuclear divisions of early embryos, evidence for pre-cellular zygotic phenotypes has been reported for only one gene – *engrailed* (*en*) [9].

The male and female pronuclei join and initiate division cycles in the central region of the egg, approximately one-third egg length from the anterior pole. During the first three mitotic cycles, nuclei remain in a roughly spherical arrangement as they expand outward and posteriorly. As the nuclei spread out along the long axis of the embryo during cycles 4–6, they form an ellipsoid with evenly spaced nuclei that are approximately equidistant from the cortex. During the ensuing pre-blastoderm divisions, the ellipsoid expands until nuclei reach the surface at the first syncytial blastoderm stage (cycle 10). These early cycles take 9–10 minutes each. Cellularization begins at cycle 14, (the cellular blastoderm stage), after three more division cycles of successively longer duration. Throughout these phases, the nuclear cycles are almost perfectly synchronized, the inter-nuclear spacing is highly regular and the movements are essentially the same in every embryo. All nuclei conform to these descriptions except for: 1) the nuclei that bud from the posterior pole at cycle 8, later to form germ cells following cycle 9; 2) approximately 100 “yolk nuclei” in the embryo interior which either do not move to the periphery or move to the interior after de-lamination from the surface; and 3) the patterned variations in mitotic cycle lengths such that for cycles 7–13, nuclei in the middle of the embryo have slightly longer mitotic cycles than nuclei in the polar regions [10], leading to apparent “mitotic waves” that originate simultaneously at the anterior and posterior poles and terminate in the mid-region [11].

Genetic studies have identified many genes whose maternal expression is required for pre-cellular (syncytial) blastoderm and cellular blastoderm formation [12], but systematic screens have failed to identify zygotic functions that are required in the embryo prior to cellularization [13], [14]. Using compound chromosomes and Y translocations, these authors generated nullo-X, nullo-2L, nullo-2R, nullo-3L, and nullo-3R embryos, but their analysis did not identify a deficiency condition that had morphological defects prior to the beginning of cycle 14.

An early period of pre-programmed development is not unique to the Drosophila embryo. For instance, Xenopus development has features that parallel the early stages of Drosophila embryogenesis: its early divisions are rapid, stereotyped and synchronous, and RNA synthesis is “low-to-undetectable”. These cell cycles are followed by a period in which the cycles slow down and are asynchronous, and gene expression is robust [15], [16]. The transition point for the dramatic changes in cell-cycle rate, synchrony and gene expression is known as the mid-blastula transition (MBT) and is called the mid-blastoderm transition in Drosophila.

The MBT model describes embryos prior to the blastula/blastoderm stage as essentially transcriptionally silent and dependent on a maternal dowry. Although widely accepted, evidence has been reported for transcription during cleavage stages in echinoderms [17], amphibians [18]–[20], fish [21] and in the Colorado beetle (*Leptinotarsa*) [22]. The Schenkel and Schnetter (1979) autoradiographic study of embryos injected with ^3^H-uridine is particularly notable and relevant: it reports clear evidence for RNA synthesis (acid-precipitable, anti-amanitin sensitive incorporation) in the female pronucleus and in subsequent pre-cellular stages except for nuclear cycle 2. However, no comparable study in this or other insect has been reported, and the functional significance of gene expression in pre-MBT embryos has not been established.

In 1985, we reported that *en* mutant embryos have an abnormal phenotype at nuclear cycle 10 [9]. For every null *en* allele that was tested, approximately one-quarter of the progeny of heterozygous parents – the embryos that are genetically *en* - could be distinguished by the abnormal position of their posterior pole cells at nuclear cycle 10. This work established that the pre-cellular *en* phenotype was zygotic and had no maternal component, but it did not identify the earliest stage that required *en* function. Pole bud formation is the first major morphological change that is visible in embryos that are viewed live with brightfield optics. The mutant phenotype indicated that *en* gene function is required at this stage, but left open the possibility that *en* is expressed and is required earlier. However, studies of younger, pre-blastoderm stage embryos were limited by the methods then available. For example, although we reported that preparations of fixed embryos from heterozygous *en* parents had some pre-blastoderm embryos with asynchronous nuclear divisions, we could not ascertain if these abnormal embryos were mutant because we lacked the ability to observe asynchronous divisions in live pre-blastoderm embryos that could be allowed to develop for genotyping. Techniques for detecting gene expression were also not sufficiently sensitive to obtain direct evidence of *en* transcripts. As reported here, the advent of PCR, genomic sequencing, RNA-seq and improved histological methods now overcome many of these technical hurdles that heretofore made early embryos inaccessible to molecular and histological study.

## Results

### The pre-blastoderm engrailed phenotype

**Figure 1:**
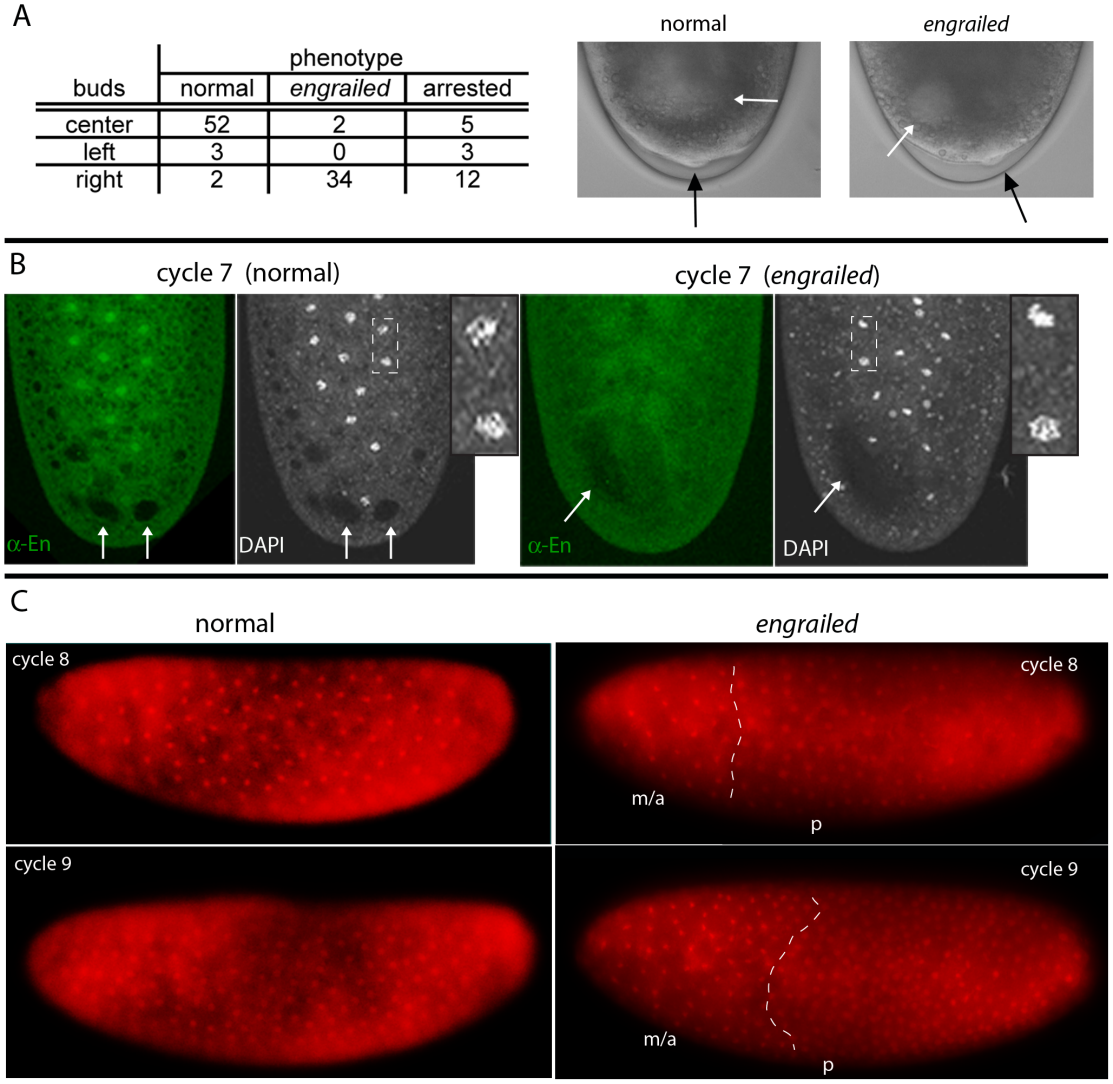
*engrailed* mutant phenotypes in pre-cellular embryos. (A) Table: Numbers of nuclear cycle 10 embryos from an *en^LA4^/CyO*×*en^LA7^/CyO* cross that were selected according to the position of their pole cells relative to the dorsal midline; most that extruded pole cells at the midline (center) developed as *en^+^*; most with pole cells offset from the midline developed as *en* mutants. The buds that appeared at cycle 8 (photos of live embryos, DIC optics) were centered in *en^+^* embryos and offset in *en* mutants (black arrows indicate buds; white arrows indicate areas of “cytoplasmic clearing”). (B) Fixed cycle 7 embryos from an *en^LA4^/CyO*×*en^LA7^/CyO* cross, stained with DAPI and anti-En antibody. All nuclei in the normal embryo (left) stain with anti-En antibody and are in prophase (inset a higher magnification view of the two nuclei in area demarcated by dashed white lines); nuclei in the *en* mutant embryo (right) did not stain with anti-En antibody and are either in prophase (e.g., lower nucleus in area inside dashed white lines) or pre-metaphase (e.g., upper nucleus). Arrows indicate areas of “cytoplasmic clearing” that are approximately centered in the normal embryo and offset in the mutant. (C) Two live embryos endowed with His-RFP imaged with epi-fluorescent optics at cycle 8 (upper panels) and approximately ten minutes later (lower panels). Orientation: anterior, left and dorsal, up. After viewing, both embryos were incubated to 24 hrs after egg laying for genotyping; embryo on left developed normally; embryo on right was *en*. Nuclei in the normal embryo are synchronous (cycle 8: late interphase/prophase; cycle 9: prophase) and evenly distributed; nuclei in the *en* mutant are neither synchronous (cycle 8: metaphase/anaphase (m/a), prophase (p); cycle 9: metaphase/anaphase, prophase) nor evenly distributed. White dashed lines approximate boundaries between regions at different cell cycle phases.

Our previous study analyzed ten *en* alleles generated from four different parental backgrounds and reported that all thirteen different transheterozygous combinations that were examined produced pre-cellular embryo phenotypes in Mendelian proportions [9]. To verify this result, we first examined living embryos from a cross of two of the lines - *en^LA4^/CyO* and *en^LA7^/CyO*. Individual embryos were oriented dorsal up and were followed with a dissecting microscope using transmitted illumination as they developed through cycles 8–10. Whereas normal cycle 10 embryos extruded pole cells centered at the dorsal midline, the pole cells of *en* mutant embryos were slightly, but reproducibly, offset from the midline. Identical results were observed in crosses of *en^LA4^/CyO* and *en^LA7^/CyO* to *Df(en^E^*), which lacks the *en* gene and most of the linked *inv* gene, but is otherwise normal [23]. The asymmetric positions of the *en* pole cells could be detected when viewed from the dorsal side, but they were not apparent when embryos were viewed from the side. This may account for the contradictory results that have been reported [13], [14]. In the experiment tabulated in Figure 1A, all embryos were not analyzed, but among 59 embryos that had pole cells symmetrically positioned at the midline, 52 developed into late stage embryos with normal cuticular patterns; they are presumed to be either *en^LA4^/CyO*, *en^LA7^/CyO* or *CyO/CyO*. Five did not progress to make cuticle and could not be genotyped, and two had fused denticle belts characteristic of *en* mutants. 54 embryos were analyzed that had pole cells that were not symmetrically positioned at the midline. Of these, 34 developed as *en* mutants, five developed as normals, and 15 did not produce cuticle. These results are consistent with our previous study that concluded that zygotic *en* function is required in pre-cellular embryos, and they were confirmed by complementation of *Df(en^E^*) with BAC transgenes that contain the *en* transcription unit amid either 45 kb or 79 kb genomic DNA (see Materials and Methods). *Df(en^E^*) animals with either transgene are viable and could not be distinguished from wildtype at the blastoderm stage.

In early cycle 8 embryos, several bulges can be seen forming at the posterior pole, and these bulges become larger and more numerous during cycle 9. In normal embryos, these bulges are centered relative to the dorsal midline, but cycle 8 embryos with bulges offset from the midline develop offset pole cells and as *en* mutants (Figure 1A). This result indicates a requirement for *en* function prior to cycle 9 (syncytial blastoderm). Several additional abnormalities were noted among the mutant embryos. First, there was a strong bias in the location of the asymmetrically positioned pole buds and pole cells of mutant embryos: in 34/36 embryos, all were on the right and none were on the left when viewed from the dorsal side. This phenotype suggests that *en* function is required to either establish or maintain left/right symmetry in the early embryo. Second, we also detected an abnormal area of cytoplasmic clearing in the posterior plasm of mutant embryos. In preparations of embryos viewed live with transmitted light (Figure 1A) or that had been fixed and stained with either DAPI or anti-En antibody (Figure 1B), we observed discrete regions near the posterior pole that appeared lighter than surrounding areas with transmitted light or darker than the surrounding background staining with fluorescent optics. Whereas these regions were approximately symmetric with respect to the dorsal midline of normal embryos, they were abnormally shaped and were not at the midline in *en* mutants. These regions could be marked with histological stains for carbohydrates (Periodic Acid-Schiff's reagent) and they were sensitive to amylase digestion (not shown), suggesting that they consist largely of glycogen. Their appearance was a reproducible (and diagnostic) feature of normal and mutant embryos.

### Cell cycle asynchrony in *en* mutant embryos

As mentioned in the Introduction, we previously reported [9] that embryos with significant mitotic asynchrony were identified in populations of embryos that included *en* mutants, but we could not assign a genotype to the abnormal embryos because available methods could not detect the asynchrony in live specimens. For instance, although nuclei (energids) are visible as early as cycle 4 with appropriate bright field optics, the resolution of such images cannot discriminate stages of the cell cycle. We took advantage of a fly line that expresses Histone:RFP in the female germline to analyze nuclear behaviors in pre-syncytial blastoderm embryos. Using an epi-fluorescence compound microscope, fluorescent nuclei were first visible at cycle 6, and we could view the embryos for brief intervals to discern the stages of the mitotic cycles without causing lethality. In a cross of *en^LA4^/CyO*×*en^LA7^/CyO* parents, most embryos had synchronous cycling and had nuclei that distributed evenly about their periphery (Figure 1C). These embryos developed normal cuticle patterns. A portion of the embryos had asynchronous divisions and regions with altered spacing; these embryos developed as *en* mutants. We did not observe differences in cycle or spacing that were greater than a single cycle and we did not recognize a reproducible pattern to the abnormalities – the regions that “lagged” varied in both size and location (Figure S1). Because stage-specific differences in nuclear morphology are revealed by the morphology of Histone:RFP-marked nuclei at some but not all stages, the asynchronous phenotypes in mutant embryos appears transiently during real time viewing.

With this evidence that cell cycle asynchrony is a characteristic of pre-blastoderm *en* embryos, we analyzed the mitotic cycles in embryos that *en*/+ parents produced. The cycle 7 embryos shown in Figure 1B are examples of a synchronous normal and an asynchronous *en* embryo. All the DAPI-stained nuclei in the normal embryo are in the prophase phase and they are evenly distributed. In contrast, the nuclei in the mutant embryo are not evenly distributed, and prophase, pro-metaphase and metaphase nuclei are present. The groups of nuclei that are at different cell cycle stages appear to map randomly to different regions in individual mutant embryos, but in each embryo, these groups of nuclei appear to be distinct. This phenotype type of “sectoring” is also a hallmark of nuclear expansion defects that have been observed in maternal myosin mutants [24].

**Figure 2:**
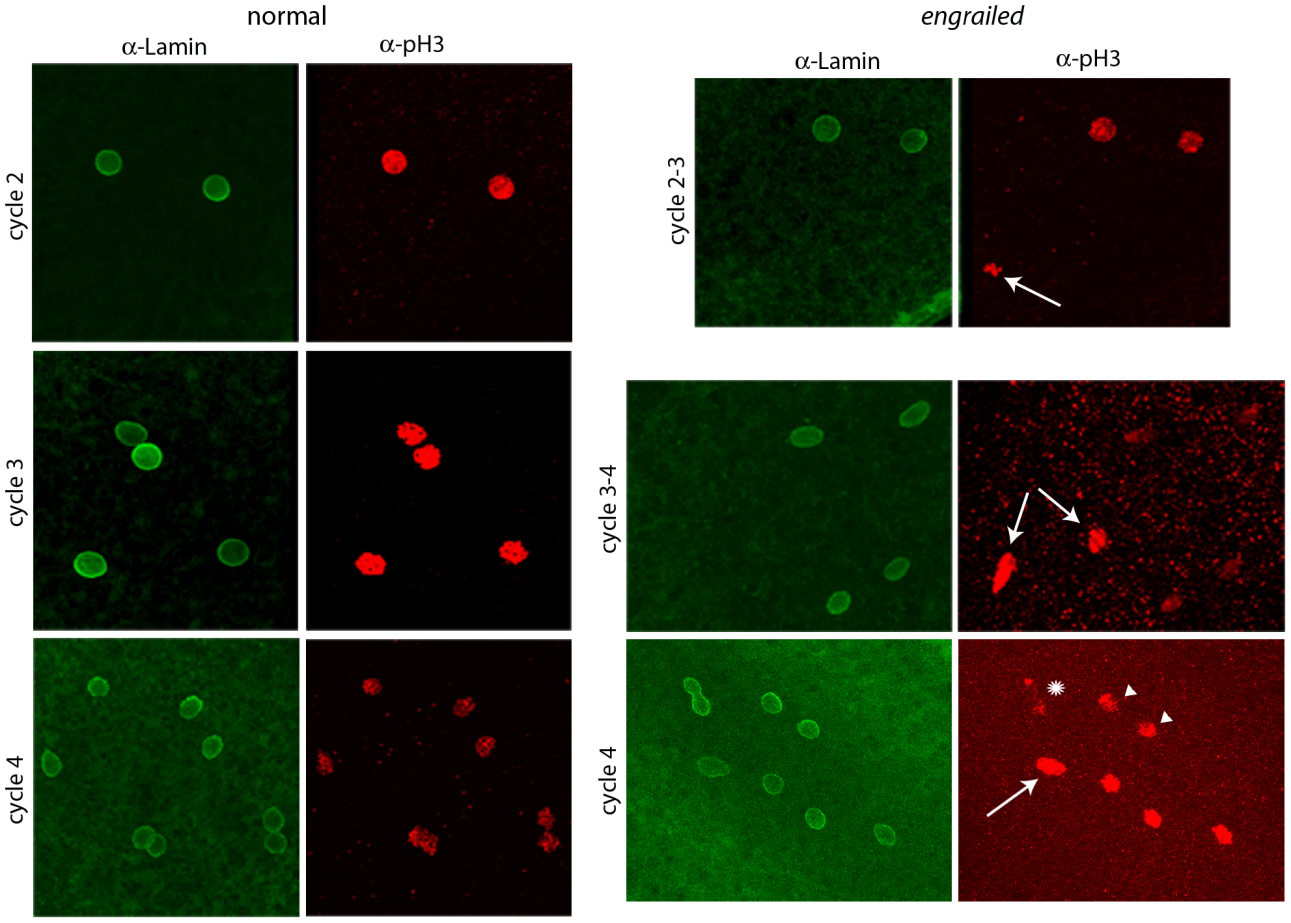
Abnormal mitotic nuclei in cycle 2–4 *engrailed* mutant embryos. Progeny from a cross of *Df(en)/+* parentals were stained with antibodies directed against nuclear Lamin and phospho-Histone 3 (pH3). Nuclei in the normals are synchronous (cycle 2: prophase; cycle 3: pro-metaphase; cycle 4: prophase/metaphase); nuclei in the *en* mutants are not synchronous (cycle 2–3: two prophase, one anaphase (arrow); cycle 3–4: four late interphase, two metaphase/anaphase (arrows); cycle 4: one prophase/metaphase (arrow), three prophase, two late interphase (arrowheads), two telophase/early interphase (abnormal staining and morphology, star).

To determine if *en* mutants exhibit mitotic asynchrony earlier than cycle 7, embryos from a cross of *Df(en)* heterozygotes were fixed and stained with antibodies directed against nuclear Lamin and phosphorylated Histone 3 (pH3). Two types of embryos could be distinguished. The majority were normal and had nuclei that were synchronous; the three examples shown in Figure 2 (a cycle 2 in prophase, a cycle 3 in pro-metaphase and a cycle 4 in pro-metaphase) are typical. The temporal resolution provided by anti-Lamin and anti–pH3 staining did not reveal any detectable variation between nuclei in any normal embryo that was analyzed. Embryos of the second type, a minority population, were not synchronous and were presumably *en*. The examples shown in Figure 2 are transitioning from cycle 2-3 (top panels) or cycle 3–4 (middle and lower panels). Embryos with asynchronous divisions were a feature only of populations with *en* mutant embryos. For example, in an experiment in which wildtype embryos were stained with DAPI and all nuclei were counted, the number of nuclei per embryo did not vary from 2^n^ in any of 461 cycle 2–7 embryos that were analyzed. In another experiment, analysis of eighteen DAPI-stained cycle 3 embryos from a heterozygous *en* cross identified sixteen with four nuclei and two with three nuclei. We conclude that the precision of the regimentation of nuclear cycling exceeds the measures that we have to distinguish variations among the nuclei in wild type embryos, but that *en* mutant embryos are unable to synchronize cycling beginning with the earliest divisions of their zygotic nuclei.

### Expression of En protein in pre-blastoderm embryos

**Figure 3:**
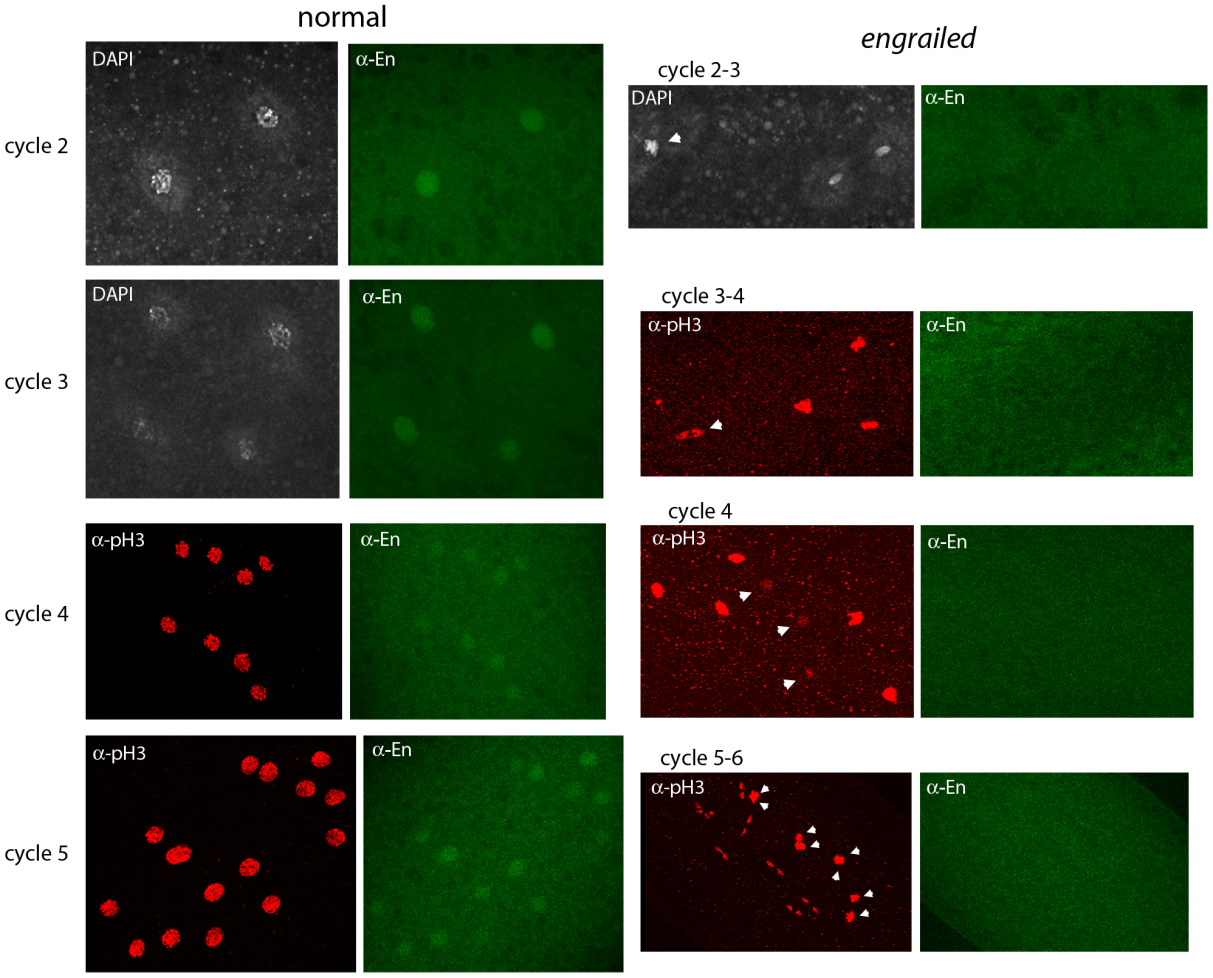
Engrailed protein is present in nuclei of pre-blastoderm embryos. Progeny from a cross of *Df(en)/+* parentals were stained with antibodies directed against phospho-Histone 3 (pH3) and Engrailed (En). Nuclei in the normals stain with anti-En and are synchronous (cycle 2, prophase; cycle 3, prophase; cycle 4, prophase/metaphase; cycle 5, prophase/metaphase (two nuclei almost superimposed in this projection image)). Nuclei in the *en* mutants did not stain with anti-En and are not synchronous (cycle 2–3: one, metaphase (arrowhead), two telophase; cycle 3–4: one, anaphase/telophase (arrowhead), three metaphase; cycle 4: three, interphase (arrowheads), five metaphase; cycle 5–6: eight metaphase (arrowheads), eight telophase).

To investigate whether En protein is expressed in pre-cellular embryos, embryos from a cross of *Df(en)* heterozygotes were stained with anti-En monoclonal antibody (Figure 3). Staining was most prominent in embryos at prophase and most prophase-stage embryos had nuclei that stained. The staining intensity was similar in all of the nuclei, and counter-staining with either DAPI or anti–pH3 antibody indicated that cell cycling was synchronous in these embryos. The normal cycle 2, 3, 4 and 5 embryos in Figure 3 are typical. A minority population of prophase-stage embryos did not stain with anti-En antibody and cell cycling in these embryos was asynchronous. The absence of detectable En protein in these abnormal embryos is consistent with the idea that the asynchronous embryos are *en* mutants and these antibody-negative embryos serve as a control for the antibody-stained normal embryos.

**Figure 4:**
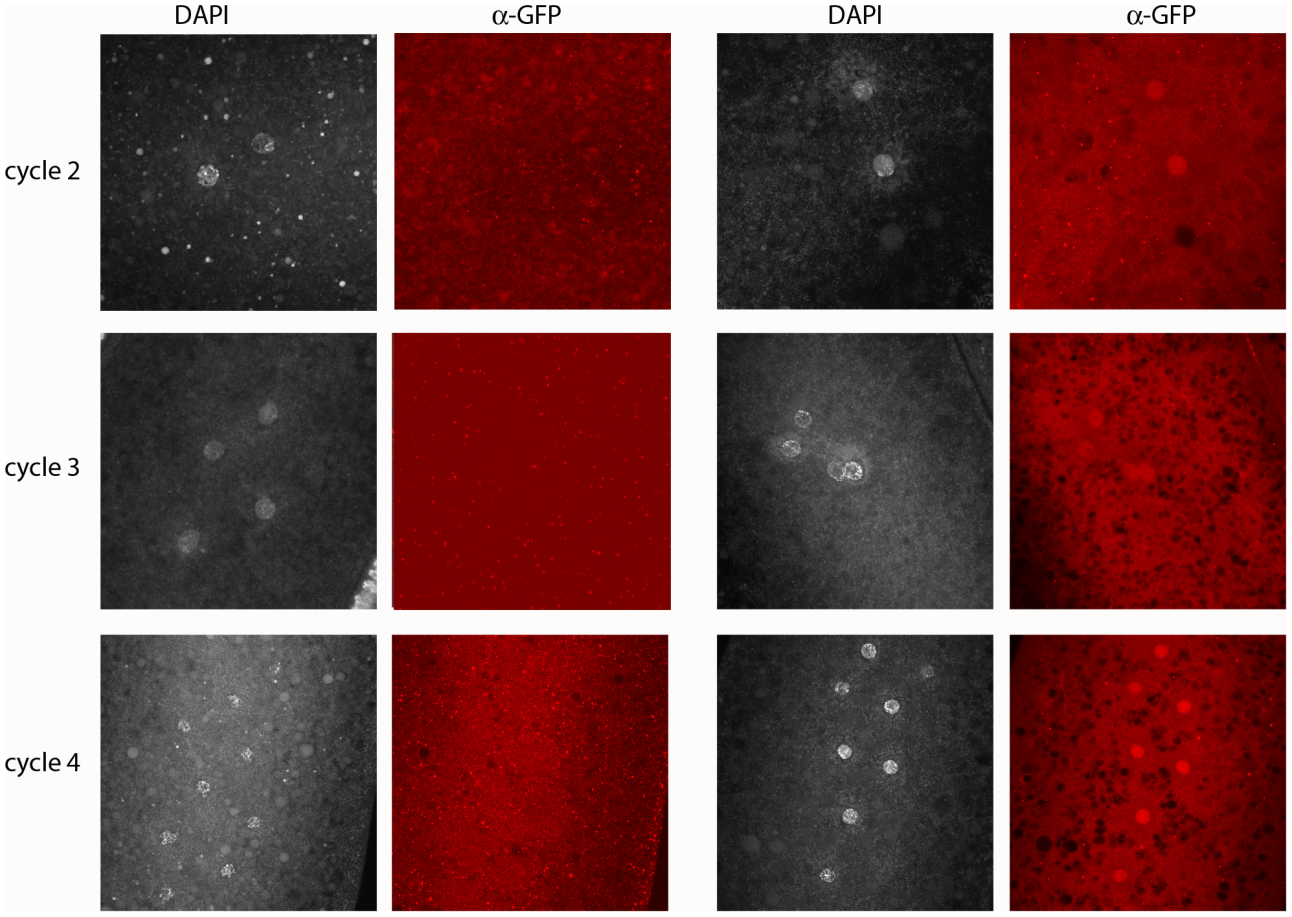
Engrailed protein is expressed by the paternal genome in pre-blastoderm embryos. Progeny from a cross of wildtype females with males carrying a 21 kb genomic BAC that had been recombineered to express an En:GFP fusion protein regulated by the *en* promoter region. Embryos were stained with DAPI and anti-GFP antibody; antigen in the nuclei of approximately half the embryos was detected by staining with anti-GFP antibody. Left two columns: GFP-negative embryos cycles 2 (prophase), 3 (prophase) and 4 (prophase/metaphase); right two columns: GFP-positive, En-expressing embryos cycles 2, 3 and 4 (all prophase).

Because the *en* gene shows no evidence of maternal effects [9], and because *en* mutant embryos did not have detectable En protein (Figure 3), the presence of the En antigen in normal cycle 2 embryos suggests that *en* transcripts had been produced and translated in the embryo - that *en* expression had begun shortly after fertilization. Additional evidence for zygotic expression was obtained by analyzing embryos that have a paternally-derived *en-GFP* BAC (Figure 4). In this experiment, embryos were stained with anti–GFP antibody. To verify that the proportion of stained embryos was consistent with normal homolog segregation, positive-staining was tabulated among 50 embryos that were at the cycle 14 to germ-band extended stages. Zebra-like stripe staining patterns were observed in 26 embryos. These presumably carried the *en-GFP* BAC and the 24 that were not staining-positive apparently did not. Pre-blastoderm embryos also stained with anti-GFP antibody, and as was observed with the anti-En antibody, the most robust staining was for prophase stage embryos. Approximately half the embryos stained, and embryos as young as cycle 2 were among them. This result supports the conclusion that *en* is expressed in the early zygote.

### *en* transcription in pre-blastoderm embryos

**Figure 5:**
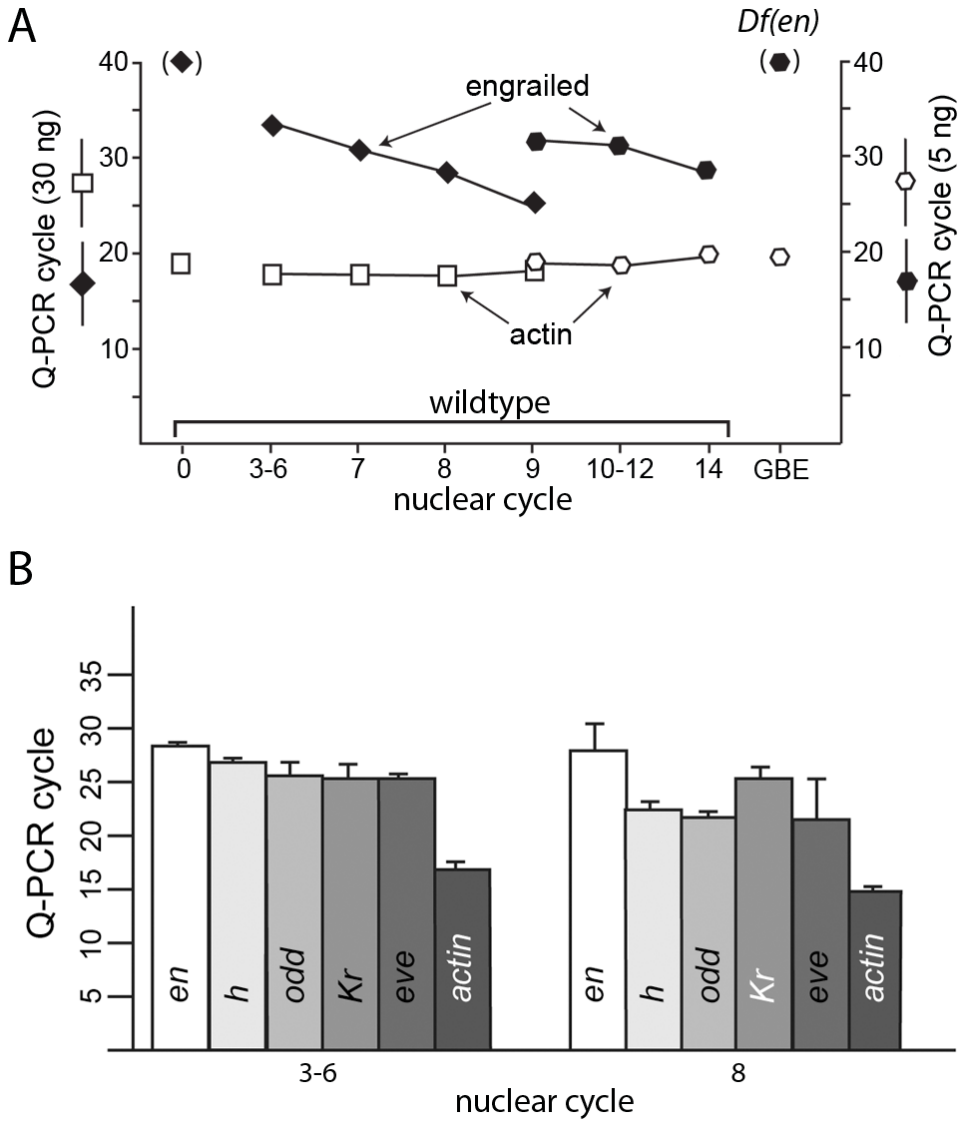
Q–PCR analysis detects transcription in pre-blastoderm embryos. (A) RNA extracted from wildtype embryos at the indicated cycles, from unfertilized eggs (0) and from *Df(en)* cycle 14 – germ band extended embryos (GBE) was used to template PCR using primers that nestle *en* intron 1 (see Table S1). No product was detected after 40 PCR cycles using RNA extracted from unfertilized eggs or from *Df(en)* cycle 10–12 embryos. Product using primers for actin transcripts was detected in all samples. (B) cDNAs prepared from RNA extracted from wildtype embryos at the indicated nuclear cycles was used to template PCR using primers that nestle an intron of *en, h, odd, Kr*, and *eve*. Amount of RNA used to prepare the cDNA templates for PCR amplification: (A) 30 ng for nuclear cycles 0–9; 5 ng for nuclear cycles 9–14 and GBE; (B) 30 ng for nuclear cycles 3–6 and 8. PCR reactions were performed in an ABI 3730 Thermocycler for (A) and a BioRad C1000 Touch Thermal Cycler for (B); the number of PCR cycles required to amplify *en* transcripts differed with the two instruments (presumably due to distinct cycling protocols), but was reproducible for each instrument.

To obtain direct evidence for *en* transcription, RNA was extracted from staged embryos for analysis. As described above, nuclei in embryos that are endowed with maternally-expressed His-RFP could be imaged at cycle 6 and at each ensuing cycle. We examined individual embryos that carry His-RFP and extracted RNA from pools of five embryos that were cycle 6 and younger (assumed to be cycles 3–6 due to the time for egg laying and preparation), and for cycles 7, 8, 9, 10–12 (syncytial blastdoderm) and 14 (cellular blastoderm). RNA was also extracted from unfertilized embryos and from cycle 10–12 *Df(en)* embryos (identified by their pole cell phenotype). PCR amplification with primers that bind to exon sequences to either side of the first *en* intron (see Table S1) did not generate product from pools of RNA from either unfertilized or *Df(en)* embryos after 40 rounds of amplification. PCR amplification did generate product from cycle 6 embryos (mean = 40 rounds) and from older embryos at successively fewer rounds of PCR amplification (Figure 5A).

**Figure 6:**
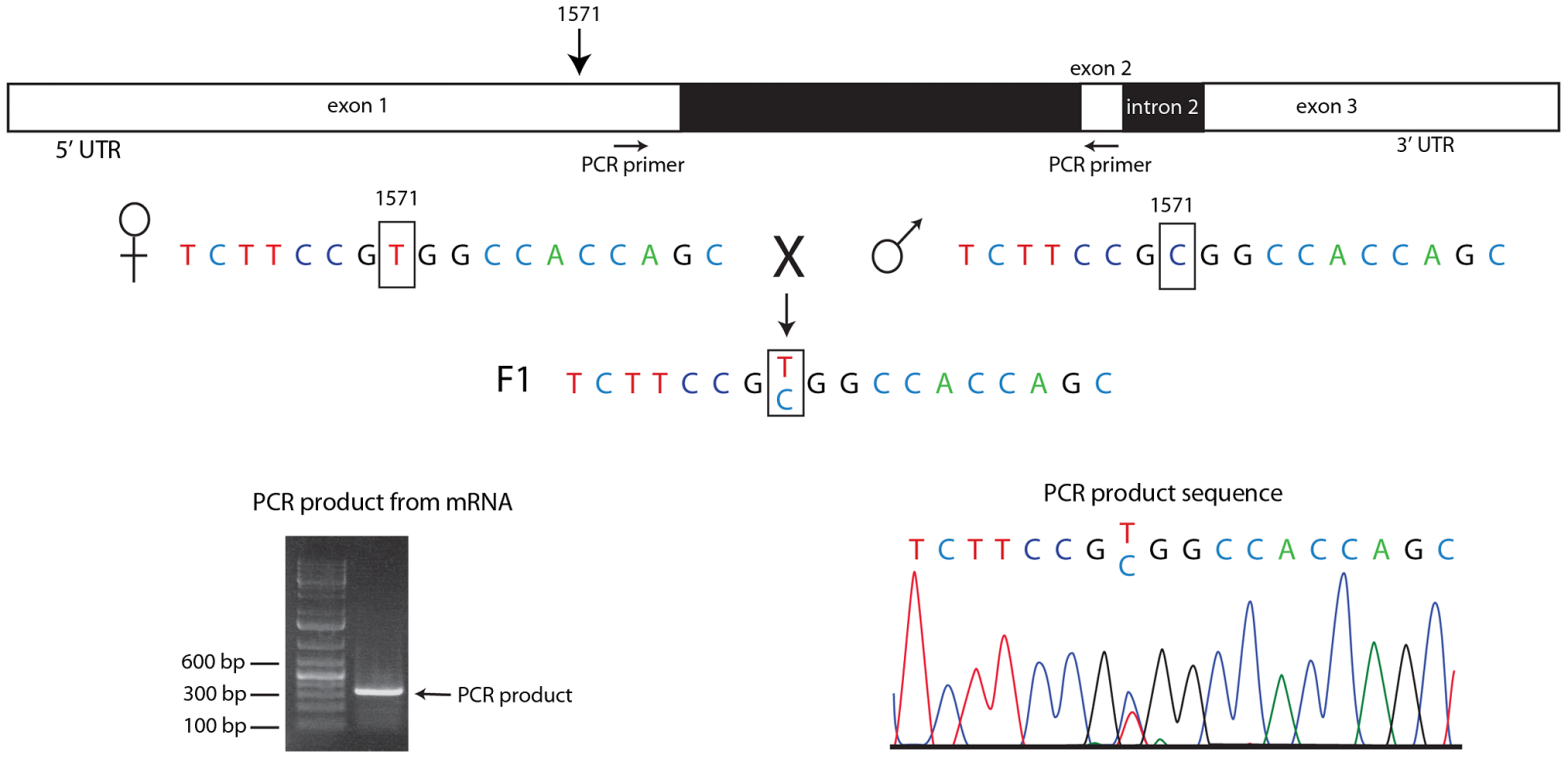
Both homologs express *engrailed* transcripts in pre-blastoderm embryos. RNA extracted from embryos heterozygous for a SNP at *en* transcript nucleotide 1571 (parental sequences shown) was amplified by PCR using primers that nestle *en* intron 1 (indicted by arrows below drawing of *en* transcript). Electrophoretic mobility of the PCR product (picture, lower left) was consistent with predicted length (336 bp) for a spliced mRNA template, and the sequence trace (lower right) indicates approximately equal amounts of both parental sequences at the 1571 SNP.

To ascertain if *en* transcripts in pre-cellular embryos are transcribed from the paternal genome, we extracted RNA from cycle 3–6 embryos that were the F1 progeny from a cross of wildtype parents whose *en* sequences differ by a single nucleotide polymorphism (SNP) at position 1571 of the *en* transcript. Position 1571 is 282 residues upstream of the first *en* intron. We used primers that bind to sequences upstream of 1571 and downstream of the first intron for PCR amplification and generated DNA of the expected size (Figure 6). The sequence of this DNA fragment revealed the presence of both the maternal and paternal SNP in approximately equal amounts. This result indicates that the alleles on both homologs were transcribed at or before cycle 6, and that *en* RNA in these pre-blastoderm embryos is a zygotic product.

### RNA–seq identifies zygotic transcripts in pre-blastoderm embryos

To investigate whether the pre-blastoderm embryo transcribes genes in addition to *en*, we analyzed sequences present in mRNA that had been isolated from individually staged cycle 3–6, cycle 7 and cycle 8 embryos. These embryos were the products of crosses of two fully sequenced, inbred lines that have polymorphisms (primarily single nucleotide substitutions) at intervals of approximately 200 base pairs [25]. Individual reads that overlap polymorphic sites can be assigned to either maternal or paternal chromosomes [26]. The method is sensitive to the number of sequence reads, relative representation in the mRNA pools, and number and distribution of SNPs in the parental lines. Strictly maternal mRNAs will have exclusively maternal genotypes, while zygotically transcribed mRNAs will have approximately equal number of reads with maternal and paternal genotypes.

Analysis of approximately 60–70×10^6^ reads for each of the mRNA pools identified reads overlapping polymorphic sites in the transcripts of 6,008 genes. Of the 5,461 genes with >100 reads overlapping a polymorphic site, maternal genotypes accounted for >90% of the sequence reads in 5,386. For genes with high representation in the mRNA pools, between 0.1 and 1% of the reads mapped to the paternal chromosome. We attribute these to sequencing errors. For approximately 70 genes, the number of sequence reads was low and the proportion of maternal and paternal SNP reads was approximately equal (Table 1). This gene set is comprised almost exclusively of genes with small transcripts that have few or no introns. We assume that the several on the list that are considerably larger (e.g., *luna* and *CG16813*) are false positives. The list includes X chromosome numerator genes *sis-A*, *sc* and *run* whose expression in cycle 8 embryos has been reported [27]. In sum, the RNA-seq data support the conclusion that embryos are transcriptionally active prior to the formation of the syncytial blastoderm.

**Table 1:** Genes identified by RNA–seq in RNA isolated for indicated stages.

GENE	Cycle 4–6	Cycle 4–6	Cycle 7	Cycle 7	Cycle 8	Cycle 8	Size	Introns
	M	P	M	P	M	P		
*luna*	6	6	12	7	13	9	128861	5
*CG16813*	2	7	6	5	24	22	996	0
*zen*	25	0	12	0	88	83	1336	1
*CG14014*			6	24	653	452	1127	0
*Brd*			9	16	291	382	530	0
*bnk*			15	15	607	563	1572	0
*sisA*			9	13	47	53	768	0
*CG15480*			24	12	153	152	501	0
*CG18269*			7	9	70	93	603	0
*pn* †			0	6	3	1	1846	1
*CG15876*			7	5	61	51	619	0
*CG8960* *			8	2	663	275	646	0
*amos* *			20	2	164	124	820	0
*sc*			5	1	112	77	1483	0
*Bro*			8	0	374	213	745	0
*SNCF*					199	109	613	0
*run*					229	162	2881	1
*m4*					174	121	733	0
*CG13716*					65	54	354	0
*l(1)sc*					37	29	1095	0
*CG14427*					92	36	1162	0
*CG13000* *					123	54	466	0
*scw*					105	88	1409	0
*eve*					153	249	1583	1
*esg*					151	95	2277	0
*scw*					8	9	1409	0
*odd* *					5	7	2000	1
*Egfr*					12	3	36.4	5
*Cpr67B*					5	7	1378	1
*w-cup*					9	1	1933	1
*CG32532* Δ			35	1	7	8	15471	8
*roX1*					15	6	3748	1
*CG7778* *					9	3	1079	1
*h*					118	130	3285	2
*CG4440* *					36	15	352	1
*CG15479* *					10	11	602	0
*CG7203*					6	2	989	1
*Bsg25A* *					37	39	1600	0
*Z600* *					4	1	300	0
*noc*					28	8	3158	1
*sog*					23	6	21971	4
*Cyp4g1*					4	2	2277	0
*tld*					30	22	3778	6
*Prm* *					5	1	7531	2
*gk*					11	5	6071	2
*hkb*					5	4	1628	1
*CG14915*					16	20	506	0
*spo*					22	21	2495	0
*tll*					58	39	2059	1
*Kr*					25	29	2918	1
*Pepck*					76	15	1845	0
*term*					32	6	1463	0
*sna*					29	25	1677	0
*fd19B*					32	22	783	0
*CG3332*					5	6	4740	7
*ftz*					21	29	1904	1
*CG14317*					68	37	1347	0
*CG13711*					89	38	743	0
*gt*					120	72	1857	1
*Doc1*					10	2	3668	5
*CG13427*					592	157	438	0
*inx3*					5	6	5075	5
*CG15634*					195	178	1444	0
*CG34214*					15	6	594	1
*Notum*					5	2	9273	3
*axo*					3	3	57714	25
*slp1*					48	47	1458	0
*D*					10	2	1711	0
*CG5973*					9	1	7764	0
*Ocho*					44	29	759	0

* not annotated transcript.

† overlap with *Nmd3*.

Δ all reads from one 500 bp region.

We did not detect *en* transcripts in the sequencing data, but we compared Q-PCR amplification times for *en* to four genes in the list of zygotic genes identified by RNA-seq: *h, odd, Kr* and *eve* (Figure 5B). Q-PCR results using templates prepared from RNA extracted from cycle 3–6 and cycle 8 embryos indicate that transcripts for all five genes were present in these pre-blastoderm embryos. The amplification with *en* primers required more cycles, indicating that *en* transcripts were less abundant than any of the others, consistent with the RNA-seq results. This suggests that deeper sequencing of these samples might have identified *en* SNPs and might identify additional genes that are expressed in early embryos.

### Abnormal development of *eve* pre-blastoderm embryos

**Figure 7:**
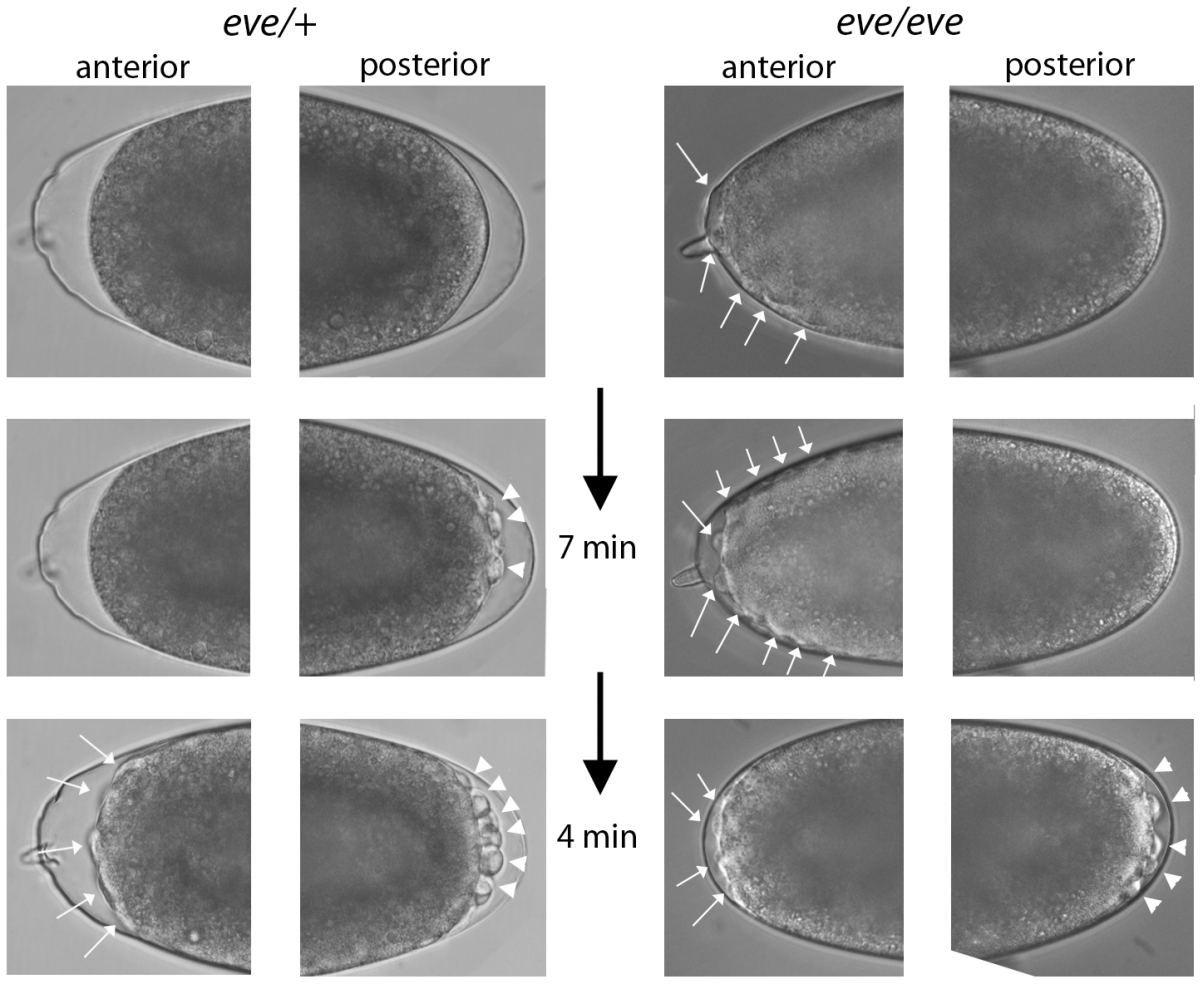
Phenotype of *even-skipped* pre-blastoderm embryos. Photographs taken at the indicated intervals of two embryos from a cross of *Df(2R)eve*/+ parents. (left panels), an embryo with normal pole cell bud formation and with no bulges at the anterior pole prior to cycle 9–10. (top), telophase cycle 8; (bottom), eleven minutes later, cycle 9–10 (right panels), an embryo with bulges at the anterior pole (top) that preceded by eleven minutes the appearance of pole buds. Left embryo developed wildtype denticle belts; right embryo developed denticle belts with the *eve* “lawn” phenotype.

Given the PCR and RNA-seq evidence for *eve* transcription in pre-blastoderm embryos, we examined progeny from a cross of *Df(2R)eve*/+ parents under a dissecting microscope looking for indications of a functional role. Normally, the appearance of posterior pole buds at cycles 8–9 and the extrusion of pole cells at cycles 9–10 are the first visible changes to the shape of the embryo; buds at the anterior pole have never been observed prior to appearance of the posterior pole cells. In contrast, some embryos from the cross of *eve* hemizygotes formed anterior buds prior to any discernable morphological change at the posterior pole (Figure 7). We selected thirty embryos with pre-blastoderm anterior buds and incubated them to late embryo stages: 24 developed *eve* denticle patterns; six did not develop sufficiently to make cuticle and could not be genotyped; no embryos had wildtype denticle belts. Pre-blastoderm anterior buds indistinguishable from those of deficiency embryos were also observed in the progeny of crosses of *Df(2R)eve/+* and *eve^3^/+* parents, and these abnormal embryos also developed *eve* denticle patterns. *eve^3^* is an ethyl methanesulfonate-induced null. These results show that zygotic *eve* expression is also essential in the pre-blastoderm embryo.

## Discussion

Embryos initiate development with a dowry of maternal transcripts, proteins and other constituents, but these stores are limited and zygotes must transition to self-sustaining production. Whereas the maternal-to-zygotic transition is universal, its timing is thought to vary in different species. For example, transcription in the mouse embryo can be detected before the first cleavage division, and at the two-cell stage, most maternal transcripts have been degraded and activation of the zygotic genome is described as “major” [28]. In contrast, the Drosophila embryo has been considered to be transcriptionally silent prior to blastoderm [6], [7], and the provisions that the egg contains have been thought to be sufficient to supply the embryo's first thirteen mitotic cycles. Moreover, studies of fly embryos that lack specific chromosome arms or entire chromosomes concluded that development is controlled entirely from maternal stores up to the mid-blastula stage [13], [14]. The present work suggests that the fly embryo may be more like the mammalian embryo than previously thought.

The statement that an embryo is transcriptionally silent is an operational conclusion based on failure to detect transcripts, and is, of course, a negative result. As methods with greater sensitivity become available, this conclusion either becomes more definitive if further experiments verify prior observations, or it must be qualified or contradicted if transcripts are detected. Similarly, assessments of mutant phenotypes depend on the resolution with which the normal can be described, and because the early Drosophila embryo has few distinctive features, our ability to distinguish or evaluate its complexities is limited. But as tools and techniques improve and the embryo's molecular and structural details are better understood, we can recognize perturbations to normal processes that could not previously be resolved.

The results presented in this paper provide evidence for functional pre-blastoderm gene expression in Drosophila. Deep sequencing of RNA isolated from pre-blastoderm embryos identified a cohort of transcriptionally active genes, and Q-PCR analysis confirmed these results for all four genes in the cohort that were tested with this method (*h, odd, Kr* and *eve*). Q-PCR was the more sensitive measure, and in contrast to RNA-Seq, it detected transcripts for these genes in embryos prior to cycle 7 (≤32 nuclei). The sensitivity of the RNA-Seq analysis was limited by the number of reads and by the fact that only those sequences that contain a polymorphism that discriminates between the maternal and paternal alleles were informative. Therefore, although the Q-PCR analysis validated the results from RNA-Seq, the cohort must be regarded as a partial list and a more complete one will require the use of more sensitive methods. The RNA-Seq cohort did not, for example, include *en*, although the evidence we obtained for zygotic production of both *en* transcripts and En protein is compelling. We suggest therefore that the abundance of *en* transcripts was below the level of detection by RNA-Seq in our experiments, a possibility that is consistent with the Q-PCR results, which also indicated that *en* transcripts are present at lower levels than *h, odd, Kr* or *eve* transcripts (Figure 5B).

The model of a silent pre-blastoderm genome is based in part on direct measures of transcription [4], [6], [7], [27] and on genetic studies (reviewed in [29]), and it seemed plausible given the rapidity of the early nuclear cycles. The interphase periods of cycles 2–9 are less than ten minutes, deemed too short for mRNA to be synthesized, processed, and translated. The finding that the zygotic genome is active prior to blastoderm therefore leads us to ask how genes can be expressed under these time constraints. We can, perhaps, look to DNA synthesis in the pre-blastoderm for a conceptual precedent. Because the length of the S phase of the early nuclear cycles is approximately one-fifth that of later cell cycles, replication in the early embryo has distinctive features (an increased number of replication orgins) that reduce the time required to replicate the genome. Although this specific mechanism is not likely to be relevant to transcription or translation, the point is that the early embryo may modify the processes of gene expression for speed and high throughput.

One key attribute of the transcripts that are expressed in the early embryo may be their small size. For example, although the *en* gene extends over more than 70 kb [30], its transcript is only 4207 nucleotides. In contrast, the transcript of its homolog *invected*, which is expressed after cellular blastoderm, is more than 32 kb. Transcripts of the *h, odd, Kr* and *eve* genes are also relatively small - 3481, 2527, 2920 and 1539 nucleotides, respectively. In addition De Renzis et al (2007) previously noted that 70% of the genes that are expressed during the first blastoderm cycles have no introns, in contrast to the estimated 20% representation of intronless genes in the Drosophila genome. Similar observations have been made in the mosquito *Aedes aegypti* [31]. Many of the genes in the cohort of pre-blastoderm genes that we identified are also intronless. Small transcript size is likely to be a pre-requisite for all genes that are expressed in fast-cycling nuclei.

A feature of Drosophila embryogenesis that may be important to early gene expression is the duration of cycle 1. Fertilization takes place in the reproductive tract of the Drosophila female, and because the time to egg-laying varies, we do not have an accurate measure of the length of the first cycle. Nevertheless, based on the proportion of cycle 1 embryos in populations of eggs that are collected at short intervals, cycle 1 can be roughly estimated to be approximately 20–30 minutes – 2–3 times longer than the ensuing cycles. The fact that the male and female pronuclei remain separate during cycle 1 and do not join to form diploid zygotic nuclei prior to the cycle 1–2 division is a further complication, but our data showing that En:GFP encoded by the paternal genome is present in cycle 2 nuclei and that *en* mutant embryos are abnormal at the cycle 2–3 division shows that embryos are competent for gene expression at these early stages.

The antibody stainings that detected En protein in cycle 2 nuclei do not allow us to calculate the amount of En protein that the embryo has made. We can nevertheless estimate minimum times needed to make an *en* transcript at the elongation rate that has been measured for Drosophila *Hsp70* (at 1.5 kb/min; [32]) or to make an En protein (at 540 amino acids/min): 2.8 min and 1 min, respectively. Although productive expression is conceivable given the length of cycle 1–2 and the interphases of the later cycles, it is possible (perhaps likely) that expression in the pre-blastoderm is more efficient than these numbers suggest. For example, RNA polymerase II elongation rates similar to those in human tissue culture cells (4.3 kb/min; [32]) would reduce the time to make an *en* transcript to less than one minute, and early embryos may tailor the protein synthesis machinery for high production levels (perhaps by increasing the load rate and density of ribosomes on mRNAs). Also, because histones and mitotic cyclins are provided maternally and are not among the genes that are expressed in the pre-blastoderm, it is possible that mRNAs from the cohort of early genes are translated throughout the cell cycle without interruption.

The phenotypes we identified in *en* and *eve* mutant embryos are perplexing given the presumptive roles of En and Eve as transcription factors. *en* mutants do not distribute nuclei normally, they do not maintain mitotic synchrony and their pole cells are not symmetrical at the embryo midline. These are curious phenotypes for several reasons. First, a number of genes have been identified that are required to break left/right symmetry, but *en* may be the only gene known that is required to establish symmetry. Second, although both the pole cell asymmetry and the abnormal behavior of the mutant nuclei are suggestive of defects to the cytoskeleton, En appears to have a strictly nuclear localization in the pre-blastoderm. Our working assumption therefore is that En functions as a transcription regulator in the pre-cellular embryo and that the cytoskeletal defects in the mutants are an indirect consequence of its loss-of-function. It is possible that En functions as a repressor to silence transcription generally and that the mitotic asynchrony is a consequence of aberrant transcripts that interfere with some aspect that is required for the rapid cell cycle; alternatively, En may regulate expression of specific targets whose mis-regulation leads to the mitotic abnormalities. Future experiments that monitor transcripts in *en* mutant embryos may reveal how En functions at these early stages.

### Perspective

The stereotypic and precisely synchronized nuclear divisions during the first hour of Drosophila development are the first recognized occurrence of pattern formation in the embryo. Although this period is also a time of complex cytoplasmic re-organization and metabolic activity, the opacity of the embryo has effectively obscured the interior of live embryos from view, and the few markers that have been available have limited studies to characterizations of nuclear behaviors and the cytoskeletal structures that orchestrate them. It is likely that these technical impediments have contributed to the failures of genetic screens to identify mutants that affect the patterned activities of the early embryo, but our finding that *en* expression is required in the second division indicates that the period of pre-cellular development can be analyzed with genetic tools. Our work shows that the fly embryo is not entirely pre-programmed, and although the phenotypes we found appear to be subtle, our functional studies have sampled only a small fraction of the zygotically active genes and therefore do not yet reveal the full extent to which control of early development is under zygotic control.

## Materials and Methods

### Fly stocks and crosses

*en^LA4^* and *en^LA7^* are EMS-induced null alleles [33]; *Df(en)* refers to *Df(2R)en^E^* [23]; histone-2A-mRFP (His-RFP; [34]); *Df(2R)eve* (46C3-46C11); *eve^3^*, a lethal amorph. *en^LA4^* and *en^LA7^* had been cleansed of linked mutations with a series of four recombinations to replace flanking DNA. Phenotypes as homozygotes, trans-heterozygotes and hemizygotes were indistinguishable. *CyO* homozygotes develop to late embryogenesis but do not secrete cuticle. BAC constructs containing 45 kb (7402 k–7446 k) and 79 kb (7386 k–7464 k) genomic sequence that straddles the *en* transcription unit were derived from RP98-9A11 and CH321-56K21, respectively [35], and were provided by Y. Cheng and J. Kassis. The *en* coding sequences include a HA tag; both constructs were inserted at attP40; both were recombined with *Df(en^E^*). The *en-GFP* BAC is described in [35].

Crosses were performed at 23°C. Embryo collections were for 30–60 minutes in the dark, and after chemical dechorionation, embryos were observed in PBS +1% Triton X-100. For phenotypic analysis, embryos were viewed with a dissecting microscope and transmitted light, and at nuclear cycle 6–7 were followed one at a time, viewing in both lateral and dorsal orientations. After nuclear cycle 10, embryos were placed on a 1% agar plate and incubated at 23°C overnight; *en^+^* and *en* mutant embryos were distinguished by ventral cuticle patterns. His-RFP embryos were collected observed under a dissecting microscope as above, and pre-cycle 6 embryos were placed on a coverslip in a drop of PBS +1% Triton X-100, transferred in inverted orientation over a depression microscope slide, sealed with Halocarbon oil and viewed with an upright epi-fluorescent compound microscope. Embryos were observed over a period of less than 30 min and subjected to irradiation for less than four minutes prior to transferring to 1% agar plates for genotyping. For molecular analysis, single embryos were selected for age and transferred to 400 µl lysis buffer (Zymo Research RNA MicroPrep kit, Cat. #R1060) on ice; five embryos were pooled for each time point.

### Immunohistochemistry

After dechorionating with 50% bleach (3 minutes), washing with PBS +1% Triton X-100, and washing with water, embryos were fixed for 25 min in 4% Ultrapure formaldehyde (Ted Pella) in 0.5X PBS, 50% heptane, devitellinized with cold methanol (30 seconds), washed 3X with 100% methanol, and 3X with PBS +0.1% Tween and 0.3% Triton X-100 (PBT) for 15 minutes. Embryos were incubated in 5% normal donkey serum (NDS; 1 hour, room temp), washed 1X with PBT, stained overnight with antibody together with 5% NDS, and stained embryos were washed 3×15 minutes with PBT, incubated 2 hours with secondary antibody in PBT + NDS and washed 5X with PBT.

Antibodies: anti-En: 4D9 [36]; anti-GFP: Sigma G6539; anti-pH3: Millipore #06-570; anti-Lamin: DHSB # ADL84.12-S

### Microscopy

Images of cycle 8 embryos were of embryos prepared and mounted as described above for embryos containing His-RFP, except with differential interference contrast (DIC) microscopy. Images of His-RFP embryos were taken with epi-fluorescence optics and processed by de-convolution. Images of antibody-stained embryos were taken with a Leica TCS SPE point-scanning confocal microscope and stacks of optical sections were combined into projection images.

### PCR and Q–PCR

PCR amplifications were carried out with the primers listed in Table S1. For Q-PCR, total RNA was prepared from five embryos with Zymo Research RNA MicroPrep kits (Cat. #R1060) and quantified by absorbance with a nanodrop spectrophotometer. cDNA was prepared using Applied Biosystem High Capacity RNA-to-cDNA kits (Cat. #4387406) starting with approximately 200 ng RNA. The Q-PCR reactions were carried out with an Applied Biosystems 3730 Thermoycler and SYBR Green, or with a BioRad C1000 Touch Thermal Cycler and SsoAdvanced SYBR Green according to manufacturers' instructions.

### RNA sequencing

RNA was isolated from the progeny of two inbred, fully-sequenced lines from the Drosophila Genetic Research Panel [25]: RAL-208 and RAL-555. Reciprocal crosses were made; RNA was from three pools, each pool from five embryos that were stages 3–6, 7 or 8. Every embryo was individually staged under a dissecting microscope. RNA was sequenced with the Illumina Genome Analyzer using protocols described in [26].

Reads from each RNA-Seq sample were mapped to the reference *D. melanogaster* genome (FlyBase release 5.27; [37], [38]) using Bowtie [39] and TopHat [40], and transcript abundances for annotated RNAs were called by Cufflinks [41]. Data from each sample were normalized so that the total expression (reads per kb of sequence, per million mapped reads; RPKM) of autosomal genes was constant. We exported reads for every gene using SAMTOOLS [42] and examined all reads for every gene to determine if they overlapped a polymorphism between RAL-208 and RAL-555 as described in DGRP release 1.0. If a read overlapped a SNP, we determined whether it was of maternal or paternal origin. The vast majority of SNP reads in genes could be assigned to maternal chromosomes. For highly expressed genes, between .1 and 1% of reads mapped to the paternal chromosome, an observation we attribute to sequencing errors. A small number of genes had roughly even numbers of maternal and paternal reads, which we attributed to zygotic transcription.

## Supporting Information

Figure S1 Abnormal mitotic nuclei in *engrailed* embryos. Four live embryos shown at two successive stage (A,B; C;D; E,F; and G,H) endowed with His-RFP imaged with epi-fluorescent optics reveal the regional variability to synchrony aberrations. Cell cycle stages are indicated in each panel and white dashed lines approximate boundaries between regions at different cell cycle phases. Orientation: anterior, left and dorsal, up.
(TIF)

Table S1 Primers used for Q-PCR and SNP reactions in Figure 5 and Figure 6.
(DOCX)
