# Supplementary figures and images for: An Essential Role for Zygotic Expression in the Pre-Cellular Drosophila Embryo
Source: PLoS Genet. 2013 Apr 4;9(4):e1003428. doi: 10.1371/journal.pgen.1003428 (PMC3616919; doi:10.1371/journal.pgen.1003428)

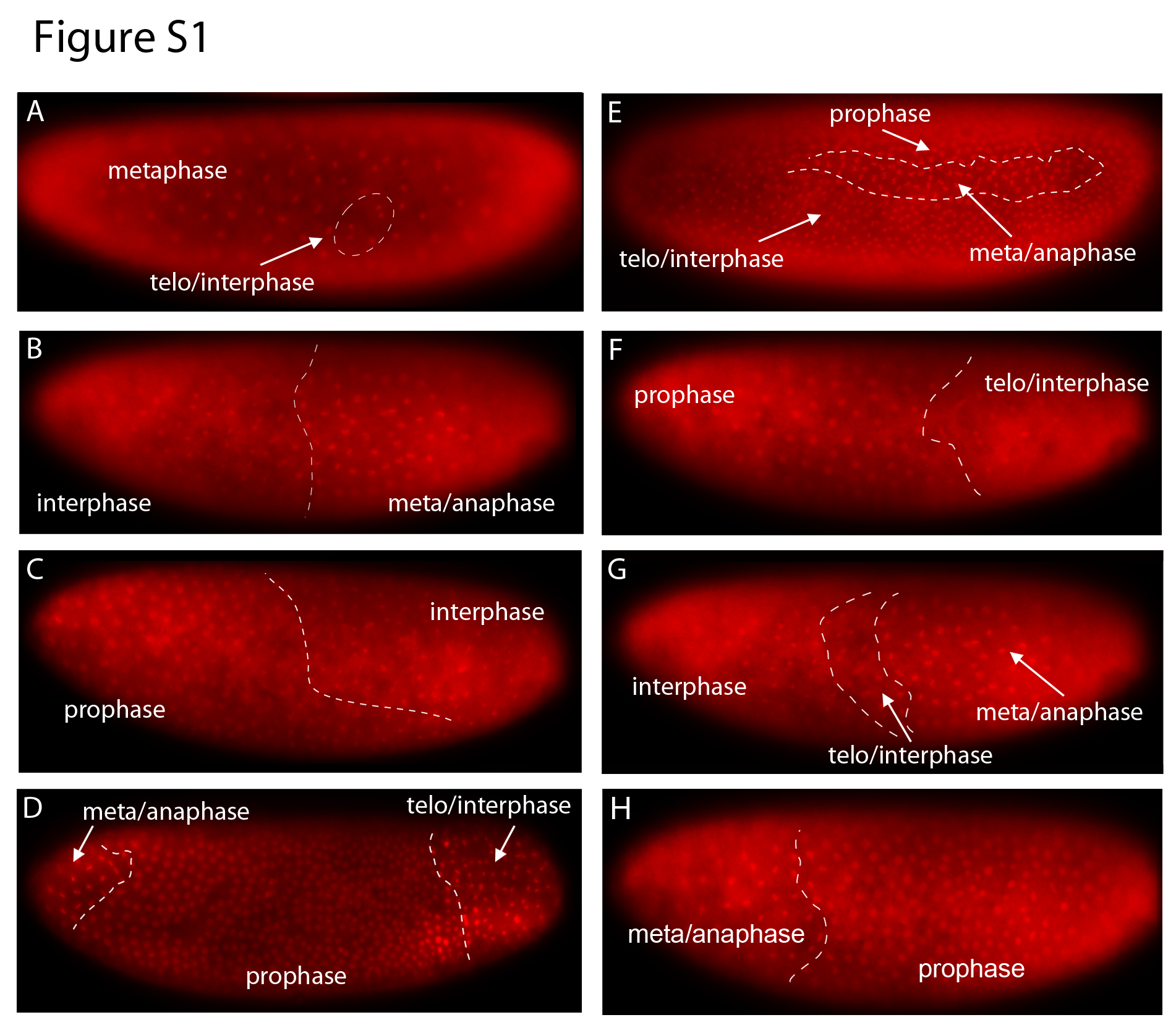

Supplement: Figure S1 — Abnormal mitotic nuclei in engrailed embryos. Four live embryos shown at two successive stage (A,B; C;D; E,F; and G,H) endowed with His-RFP imaged with epi-fluorescent optics reveal the regional variability to synchrony aberrations. Cell cycle stages are indicated in each panel and white dashed lines approximate boundaries between regions at different cell cycle phases. Orientation: anterior, left and dorsal, up. (TIF) [file pgen.1003428.s001.tif]
