# Supplementary material for: An Essential Role for Zygotic Expression in the Pre-Cellular Drosophila Embryo
Source: PLoS Genet. 2013 Apr 4;9(4):e1003428. doi: 10.1371/journal.pgen.1003428 (PMC3616919; doi:10.1371/journal.pgen.1003428)
Supplement: Table S1 — Primers used for Q-PCR and SNP reactions in Figure 5 and Figure 6. (DOCX) [file pgen.1003428.s002.docx]

|  | Forward | Reverse |
| --- | --- | --- |
| *en* (Q-PCR) | GCCTGGGTGTACTGCACCCGC | CTTCTCGTCGTTGGTCTTGTCC |
| *en* (SNP) | GCCTCGACCATTAGCAGCACCTCTTCCG | CTGTTTGGGGCGGCGGTAGC |
| *eve* (Q-PCR) | GGCCACCCAGTACGGCAAGCC | CGCGGCTGCCGTTCAAGGAG |
| *h* (Q-PCR) | CGGCCCAGCTCAAGGAGACGC | GTTGTTAATACGGGCACGTCGGCG |
| *Kr* (Q-PCR) | GAAGAATTTGTTGATGTCCATATCAATGC | GTCTAGATGAACGTCCTCTTGTTTTATGCC |
| *odd* (Q-PCR) | GCAGCAACATAACCGTGGATGACG | CTTGTCTCGCGCGACTCCTTGATC |

**Table S1. Primers for PCR**
